# Role of Metabolic Adaptation of *Streptococcus suis* to Host Niches in Bacterial Fitness and Virulence

**DOI:** 10.3390/pathogens12040541

**Published:** 2023-03-31

**Authors:** Muriel Dresen, Peter Valentin-Weigand, Yenehiwot Berhanu Weldearegay

**Affiliations:** Institute for Microbiology, University of Veterinary Medicine Hannover, 30173 Hannover, Germany

**Keywords:** metabolism, *Streptococcus suis*, fitness, transcriptome, adaptation

## Abstract

*Streptococcus suis*, both a common colonizer of the porcine upper respiratory tract and an invasive pig pathogen, successfully adapts to different host environments encountered during infection. Whereas the initial infection mainly occurs via the respiratory tract, in a second step, the pathogen can breach the epithelial barrier and disseminate within the whole body. Thereby, the pathogen reaches other organs such as the heart, the joints, or the brain. In this review, we focus on the role of *S. suis* metabolism for adaptation to these different in vivo host niches to encounter changes in nutrient availability, host defense mechanisms and competing microbiota. Furthermore, we highlight the close link between *S. suis* metabolism and virulence. Mutants deficient in metabolic regulators often show an attenuation in infection experiments possibly due to downregulation of virulence factors, reduced resistance to nutritive or oxidative stress and to phagocytic activity. Finally, metabolic pathways as potential targets for new therapeutic strategies are discussed. As antimicrobial resistance in *S. suis* isolates has increased over the last years, the development of new antibiotics is of utmost importance to successfully fight infections in the future.

## 1. Introduction

*S. suis* is a common colonizer of the upper porcine respiratory tract [1]. As almost 100% of pig farms all over the word are seropositive for *S. suis* [2], it poses a major threat to the pig industry. Especially the tonsils of healthy pigs are regarded as the natural niche of *S. suis* where the pathogen can survive and hide from the immune system [3]. *S. suis* is often considered as a commensal. However, it can also breach the epithelial barrier leading to invasive disease in its natural host. Symptoms of the disease include pneumonia, arthritis, meningitis, as well as septicemia. Moreover, subclinically infected pigs suffer from reduced weight gain leading to high economic losses in the pig industry worldwide [2,4]. The classical infection of pigs primarily occurs via the respiratory tract, either oro-nasally or via contaminated particles [5].

**Figure 1:**
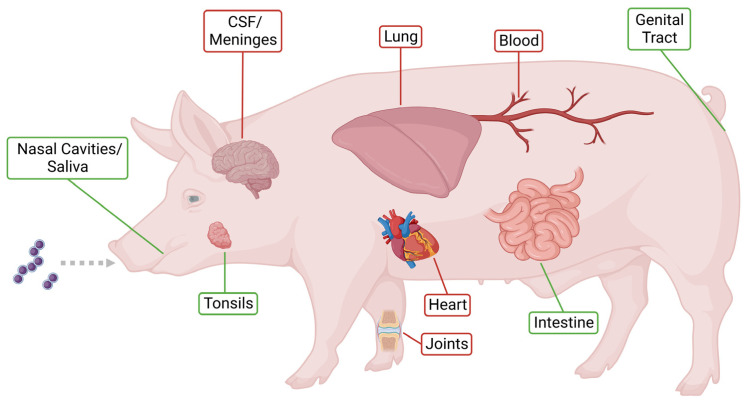
Host niches and infection sites of *S. suis* in the pig. Host niches colonized (marked in green) or infected (marked in red) by *S. suis*. Healthy colonized animals are classified as asymptomatic carriers [9,10]. Created with BioRender.com.

Importantly, *S. suis* also causes infections in humans with symptoms such as meningitis and septicemia, including streptococcal toxic shock-like syndrome [6,7]. To establish invasive infection, *S. suis* crosses the respiratory epithelium. Subsequently, *S. suis* enters the blood and disseminates within the host. Finally, *S. suis* crosses the blood–brain barrier to reach the brain and cause meningitis [3,8]. Several virulence and virulence-associated factors contribute to the pathogenicity of *S. suis* [1]. Important virulence factors comprise the capsule, the muramidase-released protein, the extracellular factor, the pore-forming toxin suilysin (SLY) as well as different adhesins and enzymes [3]. Moreover, some of them also contribute to survival in different host environments [4]. Host niches and infection sites of *S. suis* in the pig are illustrated in Figure 1.

### 1.1. S. suis Metabolism

During colonization, bacteria encounter different environmental conditions. They are faced with changes in nutrient availability and a variety of host defense mechanisms. Additionally, bacteria have to compete with residual microorganisms for resources [11]. Therefore, regulation of metabolism is key for establishing colonization and infection. Often environmental stimuli are used to control bacterial metabolism as well as pathogenicity [11]. Since nutrient-acquisition is required for successful host colonization [12], metabolic regulators were also shown to take part in virulence of different bacteria by linking environmental conditions to changes in (virulence) gene expression [12,13,14].

In addition, appropriate metabolic activity contributes to pathogen survival and infection [15,16]. During colonization and infection *S. suis* encounters host niches, such as saliva, the tonsils, the airway epithelium, the intestinal epithelium, the genital tract, joints, blood, or cerebrospinal fluid (CSF) [1,3,8,17]. The adaptation to these different body parts is accompanied by variation of metabolic gene expression [18].

The genus *Streptococcus* comprises a small genome with a size of about 2 Mbp. It is characterized by a homofermentative metabolism and mainly uses glycolysis to produce energy [19,20,21]. During the homofermentative metabolism, *S. suis* reduces pyruvate into lactate [22]. However, in the presence of glycogen heterofermentative growth with mixed acid fermentation is induced producing formate, acetate, and ethanol [23]. The annotated genome of *S. suis* suggests that it comprises several components of carbon metabolism including glycolysis as well as genes for the pentose phosphate pathway (PPP) or the Leloir pathway. Genes encoding the Entner–Doudoroff (ED) pathway are missing [20]. *S. suis* does not possess a complete tricarboxylic acid cycle (TCA) [24]. Similarly, nearly all oral streptococci lack a complete TCA and therefore, a respiratory metabolism [25]. In *S. suis* glucose is primarily metabolized via the Embden–Meyerhof–Parnas (EMP) pathway to pyruvate [20]. Moreover, phosphoenolpyruvate (PEP) plays a central role in glucose catabolism as it enables oxaloacetate synthesis by carboxylation. Oxaloacetate is an important precursor of different amino acids and other metabolites [20].

*S. suis* grows on a vast number of different carbohydrates including glucose, mannose, trehalose or raffinose as wells as maltotriose or glycogen [20,26]. To import these sugars *S. suis* mainly uses the phosphotransferase system (PTS) or ATP-binding cassette (ABC) transporters [27]. In relation to their genome size, streptococci possess a high density of carbohydrate uptake systems [28]. Subsequently, intracellular kinases phosphorylate sugars imported by ABC transporters. Thus, they can be catabolized via the EMP pathway [27].

To successfully colonize the host, it is important to keep replication “costs” as low as possible. Thus, auxotrophic bacteria may persist longer in the host as the biosynthesis of amino acid is very costly [29]. Notably, *S. suis* is auxotrophic for several amino acids in chemically defined medium (CDM), including arginine, glutamine/glutamic acid, histidine, leucine, and tryptophan [20]. For example, blood, a host site used by *S. suis* for its dissemination, is rich in glucose and free amino acids. Therefore, the auxotrophies of *S. suis* might be an evolutionary adaptation to the host environments encountered during infection [27]. Nevertheless, aromatic amino acids were shown to be crucial for *S. suis* virulence [30].

Functional groups linked to e.g., fatty acid metabolism are conserved within *S. suis* and among different streptococcal species [31]. However, functional groups linked to the biosynthesis of amino acids or nutrient uptake are less conserved representing the adaptation of different streptococci to their specific host environments [31].

### 1.2. The Respiratory Habitat of S. suis

The porcine respiratory tract represents the natural habitat of *S. suis*. Thereof especially the tonsils of healthy pigs are regarded as the main reservoir for *S. suis* [3]. Often several serotypes/genotypes of *S. suis* are present in an individual animal [32]. However, this biological niche is also inhabited by other microorganisms leading to a competitive environment [33,34]. The porcine respiratory microbiota differs between the lower and the upper respiratory tract (LRT/URT) [33]. In the URT Proteobacteria and Firmicutes predominate the natural flora. However, the genus distribution differs in the nasal and oropharyngeal cavity [35]. In the oropharyngeal cavity the most prevalent genera comprise *Streptococcus*, *Lactobacillus*, *Actinobacillus*, *Bergeyella*, *Escherichia-Shigella*, *Bacteroides*, and *Prevotella* [36]. The presence of certain streptococcal species is linked to different sites in the URT. *Streptococcus thoraltensis*, *Streptococcus pluranimalium* and *Streptococcus acidominimus* were mainly found in the nostrils, whereas *S. suis*, *Streptococcus porci* and *Streptococcus hyointestinalis* were primarily isolated from tonsil samples [33]. To survive and integrate themselves in these mixed microbial communities, streptococci generate different extracellular factors [37]. In addition, these communities are highly competitive for space and nutrition. Therefore, streptococci must cope with inhibitory molecules of other bacteria such as bacteriocins or toxins, compete for adhesion sites and additionally evade the host defense system [38]. As these bacteria-rich environments on the respiratory epithelium differ substantially from sterile body parts, e.g., the blood stream or the brain, streptococci need to rapidly adjust their metabolism upon the initiation of infection [27,39]. *S. suis* has evolved different mechanisms to assert itself in the respiratory tract. The multidrug-resistant strain WUSS351 comprises different antimicrobial systems that the pathogen also uses for bacteriocin production and release [40]. These bacteriocins constitute an important strategy to combat other microorganisms [41,42,43]. The bacteriocin Lcn351 of strain WUSS351 is especially active against two different serotypes of *S. suis* but was also able to reduce the growth of *Bacillus subtilis* [40].

Respiratory disease poses an economic threat to the pig industry worldwide. Due to its multifactorial character, pneumonia in pigs is often termed porcine respiratory disease complex (PRDC) [44]. Factors contributing to the PRDC are environmental conditions such as overcrowding, bad air quality and poor hygiene, animal specific factors like age or immune suppression as well as infection with different viral and bacterial pathogens. These pathogens can be categorized into primary pathogens leading to prior damage of the respiratory epithelium and secondary or opportunistic agents benefiting from these lesions. *S. suis* is regarded as a classical secondary pathogen [44]. Apart from *S. suis*, *Actinobacillus pleuropneumoniae* plays a notable role in the PRDC [45]. *S. suis* and *A. pleuropneumoniae* were shown to form mixed biofilms in vitro [46]. Biofilm formation represents an important survival strategy of bacteria to combat the host immune system or antibiotic treatment [47]. Both *S. suis* and *A. pleuropneumoniae* exhibited a higher resistance towards antibiotics as well as an upregulation of virulence related genes. However, growth of *S suis* was negatively affected in the presence of *A. pleuropneumoniae*, whereas growth of *A. pleuropneumoniae* was not affected in the presence of *S. suis*. All in all, co-culture in mixed biofilms seems to induce cooperative behavior of the pathogens which might help to establish and maintain infection in vivo [46]. 

Additionally, commensal bacteria in a microbiome or biofilm can benefit from their neighbor microorganisms. Whereas anaerobic bacteria grow in the core of a bacterial community, aerobic, and facultative aerobic taxa live in the periphery. Furthermore, these communities share nutrients and different metabolites as consumers and producers stay close to each other [48]. Knowledge of specific effects on *S. suis* metabolism in microbial communities is scarce and needs to be further investigated in the future.

## 2. Transcriptomic Response of *S. suis* to Host Environments

*Streptococcus suis* infection can lead to septicemia, endocarditis, pneumonia, arthritis, peritonitis, and meningitis both in pigs and humans [2,4,6,7]. Adaptation mechanisms can be revealed by analyzing the transcriptomic and proteomic responses of *S. suis* in the various host niches. Transcriptomic analysis of *S. suis* has been conducted in various models including in vivo, ex vivo in blood and spinal cord, in primary and immortalized cell lines and modified cell culture systems such as blood–cerebrospinal fluid model systems. The results from these studies pointed out that there are specific adaptations of *S. suis* to the different in vivo niches, which influence virulence and/or survival [18,49,50,51]. In addition, host responses, mainly of inflammatory cytokine responses, have been reported [52,53]. 

Furthermore, comparative transcriptomic analysis of *S. suis* epidemic strains revealed that genes linked to methionine biosynthesis and uptake as well as genes related to adhesion and immune evasion contributed to the increased pathogenicity of epidemic isolates. The upregulation of amino acid metabolism seems to be crucial, especially for the early interaction of epidemic strains with the host [54]. The infection with epidemic strains is characterized by a strong immune response of the host resulting in excessive inflammation [55].

### 2.1. Transcriptional Response of S. suis after In Vivo and In Vitro Infection

Experimental infection of piglets with *S. suis* revealed significant differential expression of putative regulator genes from bacteria isolated from the infection materials compared to those grown in vitro in Todd–Hewitt broth (THB) medium [50]. Most of the identified regulators in this study, including CodY and CiaR are predicted to participate in metabolism and transport as well as pathogenesis and virulence. Previous studies with mutants of *codY* (in a mouse infection model [56]) and *ciaR* (in macrophage, mice and pig infection models [57]) have indicated attenuation of mutant strains, strengthening the speculation that in vivo induction of these regulators enhances the resistance against phagocytosis and antimicrobial peptides promoting survival of the pathogen in the blood, the crossing of the blood–brain barrier as well as colonization of the meninges [50]. Furthermore, *covS*, a gene coding for one protein in the CovRS regulatory system of the global repressor of virulence and colonization in many pathogenic bacteria [58,59], were down-regulated in vivo compared to in vitro in THB. Taken together, the differential regulation of these and, other repertoires of regulators are predicted to be used by *S. suis* for adaptation in different in vivo niches [50,60]. 

In a recent study, where transcriptional analysis was conducted in *S. suis* after intranasal infection of piglets showed that virulence of *S. suis* plays a role in the host immune response in different in vivo niches [61]. In this study, they compared innate immune responses after intra-nasal infection of colostrum deprived piglets by *S. suis* serotype 2, virulent strain 10 (S10) and avirulent T15. Accordingly, they observed slight changes in the expression of genes coding for antibacterial innate immune response in blood, with S10 having an earlier response compared to T15, a more sustained transcription of inflammation related genes such as interleukin 1 beta (IL1B), IL1A, and interferon regulatory factor 7 (IRF7) in the nasal swabs of S10 infected piglets. However, most of the differential gene expression in trachea, lung and associated lymph nodes was observed in piglets infected with the non-virulent T15 strain. Therefore, the authors concluded that the sustained immune response at the lymph nodes during infection with the less virulent T15 strain might have contributed to the rapid control at the site of infection. On the contrary, the virulent strain prevented robust lymph node response thereby maintaining the bacterium at the site of infection, which continues to elicit inflammatory mediators [61]. The clinical outcomes could be influenced by several factors including environment, host or bacterial virulence. 

*S. suis* is involved in the infection of the central nervous system, with lesions on the choroid plexus [62,63,64]. Research has been conducted on the transcriptomic analysis involving the cells of the central nervous system, such as the choroid plexus cells [51,65,66,67]. Accordingly, choroid plexus cells of porcine and human origin, challenged with *S. suis* respond via cytokine and chemokine gene expression and protein secretion. Moreover, transcriptomic analysis of porcine alveolar macrophages, primary porcine choroid plexus epithelial cells (PCPEC) and THP-1 monocytes infected with *S. suis* revealed overrepresentation of genes involved in the host immune response, apoptosis or programmed cell death, as well as signal transduction pathways [51,52,53].

### 2.2. Transcriptomic Analysis of S. suis in Blood and Cerebrospinal Fluid

As *S. suis* has a wide range of serotypes and strains, establishment of disease requires virulence and metabolic activity of the involved strain in the respective host environment [18]. To reveal the adaptation mechanisms of *S. suis* to different host niches, Koczula et al. performed transcriptomic analysis of *S. suis* grown in blood and CSF and compared their results with the bacterium grown in THB medium. Surprisingly, distinct differences were observed in gene ontologies of the differentially expressed genes in these in vivo niches. Genes coding for carbohydrate transport and metabolism were differentially expressed in *S. suis* grown in blood suggesting a lack of glucose as the main sugar source in the bloodstream, whereas genes involved in the production of branched-chain and aromatic amino acids were differentially expressed in CSF to fight low amino acid concentrations. Many amino acids were reduced ten-fold in porcine CSF in comparison to serum [18]. Although the central carbon metabolism is conserved in CSF and blood, biosynthesis of amino acids varies, e.g., the production of isoleucine is increased in CSF [20]. 

In another study by Wu et al., it was reported that genes associated with the synthesis of capsular polysaccharide (CPS) were significantly upregulated in contrast to downregulation of these genes in CSF [49]. This shows a mechanism of how *S. suis* evades the host defense as well as its adherence and invasion mechanisms in the different host niches. 

Many sRNAs have been identified in different streptococcal species, such as *Streptococcus pyogenes* [68,69], *Streptococcus pneumoniae* [70,71], *Streptococcus mutans* [72], and *Streptococcus agalactiae* [73]. Interestingly, small RNAs (sRNAs) as regulators of virulence in *S. suis* were identified for the first time using transcriptomic approaches in blood and CSF [49]. In that study, 29 sRNAs were identified in *S. suis*, of which some are involved in the regulation of polysaccharide capsule synthesis. 

The close link between metabolism and virulence is not only mirrored in the transcriptional data of *S. suis* but is also reflected in the genome organization of this pathogen. The pathogenicity of bacteria is often associated with a reduction in genome size. Accordingly, *S. suis* disease isolates comprise a smaller genome than commensal strains [74]. Endosymbionts or mutualists often lose their metabolic genes as they highly rely on the host nutrient supply [75,76]. However, in *S. suis* the reduced genome size in virulent strains is not associated with a loss in metabolic genes. In contrast, pathogenic *S. suis* strains even had more metabolic genes than commensal ones [74].

## 3. *S. suis* Metabolism, Biological Fitness and Virulence

Metabolism regulation is key for pathogen survival and virulence [11,12,13,14]. In the following chapter we focus on the role of metabolism in different biological processes. Firstly, we focus on metabolism and its relevance for biological fitness as well as the close link between metabolism regulation and virulence gene expression. Secondly, we will focus on the effects of co-infections with other pathogens in the respiratory tract. Finally, we describe the role of *S. suis* metabolism in antibiotic resistance.

### 3.1. Catabolite Control Protein A (CcpA)

Nutrient-acquisition is the main goal for all living organisms, including pathogenic and commensal bacteria [77], as well as a prerequisite for successful colonization and infection [12]. Where available, glucose is the preferred carbon source of *S. suis* and it is essential for replication and survival in the host [27]. In general, blood contains high levels of glucose compared to other body parts [27,78]. One of the main regulators of glucose metabolism in *S. suis* is the catabolite control protein A, (CcpA). Furthermore, CcpA also contributes to bacterial fitness of *S. suis*. Similarly, the amylopullulanase (ApuA) and the arginine deiminase system (ADS) contribute to this. In the following, these regulatory systems are explained in more detail.

To ensure the uptake of the preferred carbon source, bacteria make use of a regulatory mechanism called Carbon Catabolite Control (CCC) [22,77]. This process can be divided into Carbon Catabolite Repression (CCR) and Carbon Catabolite Activation [77,79]. When the preferred carbon source is available, CCR downregulates the expression or activity of genes involved in the usage of secondary carbon sources [77]. Thereby, the bacteria can optimally utilize the available nutrients, thus competing successfully with other microorganisms [80]. CcpA controls CCR by binding to specific motifs in the promotor region, so-called cis-acting catabolite response element (*cre*) sites [81,82]. CcpA plays a key role in the metabolic adaptation of gram-positive bacteria including many pathogenic streptococci, e.g., *S. suis* or *S. pneumoniae* [24,83]. In *S. suis ccpA* expression is constitutive [13]. CCR can be classified as a carbon source intake mechanism contributing to carbon metabolism in general.

Willenborg et al. investigated the role of CcpA during growth with glucose consumption by analyzing the transcriptome of *S. suis* serotype 2 strain 10 and its *ccpA*-deficient mutant. Most of the differentially expressed genes encoded for transcriptional regulation, metabolism, and other unknown functions. Some of the affected genes seemed to be part of CCR regulation, as their expression increased in the *ccpA*-deficient mutant. These were mainly related to carbohydrate metabolism or carbohydrate and amino acid transport, e.g., the arginine deiminase system (ADS, *arcABC*) or the glycogen synthase cluster (*glgCAB*) [24]. CcpA plays a major role in glycolysis and takes part in galactose utilization. However, it is not involved in the PPP [24]. Many affected genes were not directly controlled by *ccpA*. Therefore, their regulation in vivo might also include the activity of cofactors [24].

Tang et al. also investigated the effect of *ccpA* on CCR [84]. Their study showed that *ccpA* is involved in the repression of α-galactosidase and β-glucosidase activities. The deletion of *ccpA* reduced the repression of these enzymes but did not alter their sugar utilization pattern. However, the activity of the α-glucosidase was not significantly affected in the *ccpA*-deficient mutant, suggesting the contribution of other factors to CCR e.g., potential phosphotransferase systems [84].

The group of Lang et al., applied gene expression profile analysis, metabolomics, as well as proteomics, to investigate the role of *ccpA* in *S. suis*. Their studies underlined an involvement of CcpA in sugar, amino acid, nucleic acid and fat metabolism as *ccpA* activity alters the concentration of certain metabolites [85,86]. A decrease in succinic, aspartic, and citric acid concentrations changed glucose availability and therefore, affected *S. suis* metabolism regulation [87].

As already mentioned, there is a close relationship between metabolism and virulence in bacteria. Pathogens often concatenate/combine regulation of expression of metabolic with virulence genes as it saves energy [88]. In gram-positive bacteria this is achieved by three main global regulators, CcpA, CodY and Rex [88]. CcpA and CodY were shown to be involved in *S. suis* capsule expression and virulence [13,22,56]. Many genes associated with virulence were downregulated in the *ccpA*-deficient mutant such as, suilysin, opacity factor, surface antigen one or the capsule synthesis cluster [13,84]. Interestingly, the expression of the virulence factor *arcB* was higher in the knockout strain [13]; *ArcB* encodes an ornithine carbamoyltransferase and is part of the ADS involved in pathogen survival and fitness [89,90]. The ADS system is described in more detail in Section 3.3.

The capsule of *S. suis* protects the pathogen from being phagocytosed [91,92]. Depending on the host environment, capsule synthesis is either enhanced to act as a protection against the host immune system, e.g., in the bloodstream, or decreased to facilitate adherence to epithelial barriers and subsequent invasion of the tissue [8]. In host environments containing high glucose levels, capsule expression depends on *ccpA*. Gene expression analysis of the *ccpA*-deficient mutant revealed downregulation of capsule and sialic acid synthesis [13,84]. The *ccpA*-deficient mutant of *S. suis* showed an attenuated phenotype with reduced survival in a phagocytic assay which might be linked to the lower capsule expression [13]. Former studies have already shown that an unencapsulated *S. suis* mutant showed reduced colonization capacity and resistance to phagocytosis as well as attenuated virulence in a mouse infection model [91,93]. As the capsule is one of the main factors for mediating colonization, invasion, and resistance to host defense mechanisms, its regulation is of utmost importance for establishing infection [3]. Therefore, *ccpA* plays an important role for the evasion of host immunity by contributing to phagocytosis resistance via capsule expression [13]. 

Accordingly, the deletion of the global regulator CodY also resulted in a reduced resistance to phagocytosis [56]. A *codY*-deletion mutant displayed a reduced capsule thickness as well as an inhibited expression of sialic acid genes. This was also reflected in the altered capsule composition showing a reduced amount of sialic acid content [56]. CodY regulation activity is linked to amino acid availability as well as stress [50,94]. Its expression was upregulated in *S. suis* isolated from the bloodstream or the brain. Therefore, the authors suggested that CodY supports resistance to phagocytosis as well as antimicrobial peptides which is a prerequisite for survival and colonization of these host environments [50]. Furthermore, both the adhesion and invasion capacity to and into endothelial cells as well as the virulence in a mouse infection model decreased in the *ccpA*-deficient mutant [84]. Thus, CcpA itself is regarded as a virulence factor [13].

Interestingly, Zhang et al. observed a comparable phenotype in an *hp0197*-knockout mutant of *S. suis* serotype 2 strain 05ZY [95]. HP0197 is a surface protective antigen without sequence homology to other proteins [96,97]. The *hp0197*-deficient strain showed an attenuated phenotype in mice and pig infection experiments, a decreased resistance to phagocytosis as well as a similar pattern of differentially expressed genes as a *ccpA*-deficient strain in other studies [13,95]. However, *ccpA* was not downregulated in the *hp0197*-deficient mutant. Phosphorylated HPr, a phosphocarrier protein, is an important co-effector for CcpA binding to *cre* sites [98]. HPr isolated from Δ*hp0197* exhibited a weaker binding in combination with CcpA in comparison to wild-type HPr indicating reduced phosphorylation of HPr in the mutant [95]. Therefore, these studies underline the importance of CcpA for bacterial virulence. Similar findings were observed in other pathogenic streptococci. *CcpA*-deficient mutants showed an attenuated phenotype in mice infection experiments [83,99]. Wen and Burne showed that in *S. mutans* CcpA is essential for the formation of biofilms [100]. Furthermore, CcpA is involved in the colonization and survival of *S. pneumoniae* on the respiratory epithelium [83]. In *S. pyogenes ccpA* activates the transcriptional regulator Mga which is involved in the expression of virulence genes [101]. Additionally, the expression of virulence factors regulated by *ccpA* is dependent on the nutrient availability in the environment [77]. 

To evade the host immunity and to overcome nutrient starvation, *S. suis* is able to form protective biofilms [102]. However, biofilm formation has also the disadvantage of reduced pathogenicity reflected in decreased metabolism and virulence gene expression as well as inhibition of the efficacy of bacterial toxins as they are trapped in the extracellular matrix [103]. Recently, Bulock et al. investigated the effects of *codY* and *ccpA* deletion in *Staphylococcus aureus* on biofilm formation [104]. The *ccpA*-deficient mutant showed impaired biofilm formation, whereas the *codY*-deficient mutant formed a robust biofilm structure. In the *ccpA-codY*-double knock-out strain, the overall biofilm mass was reduced indicating a linkage between central metabolism and biofilm formation [104]. Similarly, *ccpA* deletion in *Streptoccocus gordonii* led to impaired biofilm formation [105]. To the best of our knowledge, the effect of *ccpA* deletion on biofilm formation in *S. suis* has not been investigated so far. However, based on results published for other Streptococci [104,105] a similar effect is likely.

**Figure 2:**
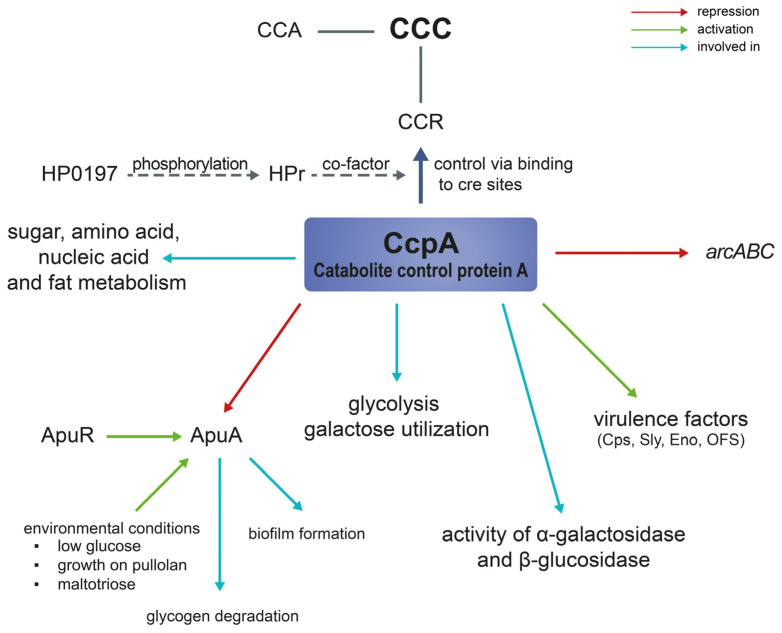
Carbon catabolite repression and the role of catabolite control protein A (CcpA) in *S. suis*. *CcpA* controls the CCR in *S. suis* via binding to *cre* sites [22,24]. HPr an important co-factor of this binding is phosphorylated by the protein HP0197 [95]. CcpA is involved in diverse metabolic pathways, the expression of virulence factors and certain enzymes such as the α-galactosidase [13,24,84,85,87]. In addition, it takes part in glycolysis and galactose utilization. *CcpA* represses the *arcABC* operon [24] and the expression of *apuA*. ApuA is regulated by ApuR and primarily induced during growth on pullulan or under glucose-deprived conditions. It plays a role in glycogen degradation as well as biofilm formation [22,23].

Figure 2 illustrates the role and functions of *ccpA* in *S. suis* metabolism as well as virulence. In summary, *ccpA* acts as a carbon catabolite repressor in *S. suis* but is also involved in many other cellular functions and can indirectly influence transcription [24].

### 3.2. Amylopullulanase (apuA)

Although glucose is the preferred carbon source of *S. suis* [27], the pathogen needs to adapt to varying sugar availability in different in vivo situations. On the one hand, glucose is present in the oropharyngeal cavity, but at varying concentrations, which are affected, e.g., by uptake of food. After feeding, glucose concentrations drop due to the direct use of this carbon source either by the host or the resident microflora [22,106]. On the other hand, the concentration of starch α-glucans in the oral cavity is much more stable and a substantial part of animal feed [22,107]. Ferrando et al. compared metabolism and virulence gene expression during *S. suis* growth on either glucose or α-glucan starch (pullulan). They showed that both the expression of an amylopullulanase, *apuA*, and the pore-forming toxin suilysin *(sly*) increased upon growth on pullulan. 

Suilysin (SLY) is a member of the group of cholesterol-dependent cytolysins (CDC) which can be found in many gram-positive bacteria [108]. SLY is considered a virulence-associated factor due to its essential contribution to the pathogenesis of *S. suis* [109,110]. SLY is cytotoxic to a variety of different cell types including epithelial cells, endothelial cells, phagocytes, red blood cells or more complex cell culture models, e.g., air–liquid interface cultures or precision-cut lung slices (PCLS) [108,111,112,113,114,115]. In addition, SLY enhances both adherence and colonization in the PCLS system [114]. Therefore, SLY participates in both the pathogenic as well as the commensal phase of *S. suis*.

Both *apuA* and *sly* contain a conserved *cre* motif in their promoter region suggesting repressed transcription during growth on glucose. The authors also identified a potential *cre* site in many other virulence genes differentially expressed in pullulan compared to glucose, encompassing the capsular polysaccharide [22]. The direct binding of CcpA to both the *sly* promotor and the capsule synthesis cluster was confirmed by Willenborg et al. [24]. Moreover, the expression of *apuA* was induced by maltotriose. The authors suggested that *apuA* might be activated by the putative transcriptional regulator ApuR and repressed by CCR mediated via *ccpA* [22]. Accordingly, the transcription of *apuA* and *sly* was higher in body sites containing less glucose than the blood, e.g., the brain, heart and joints facilitating colonization, invasion, and the use of alternative carbohydrates. Though, these findings have yet not been proven in vivo, they may contribute to new therapeutic strategies, e.g., adaptation of feed composition or blocking of certain enzymes needed for starch degradation [22]. The induction of the expression of the ADS and *sly* at low glucose levels underlines the importance of nutrient availability for *S. suis* pathogenicity [13]. At low glucose concentrations *sly* might be relieved from *ccpA* regulation explaining the increased expression during growth on pullulan [22]. Furthermore, the authors revealed that the adherence and invasion capacity of *S. suis* to newborn pig tracheal (NPTr) cells increased upon growth in DMEM supplemented with pullulan instead of glucose. The elevated expression of *sly* might contribute to this observation, as the toxin has already been shown to promote invasion to host cells [93].

Additionally, the increase in *sly* expression resulted in a higher hemolytic activity of bacteria when grown in pullulan. Although, the increased expression of *sly* probably resulted from the missing CCR rather than a starch/pullulan specific effect [22]. 

Furthermore, Tan et al. investigated the effects of exogenous glycogen utilization on *S. suis* pathogenicity. For this, the authors constructed an *apuA*-deficient mutant and compared its growth on glycogen with the wild-type strain. Inactivation of *apuA* led to a switch from homofermentative to heterofermentative metabolism inducing mixed-acid fermentation [23]. Supplementation of the media with glycogen resulted in an increased hemolytic activity of the pathogen, which is in accordance with the induced expression of *sly*. However, the deletion of *apuA* decreased *sly* production. Furthermore, the presence of glycogen induced a higher adhesion and invasion capacity whereas the deletion of *apuA* had the opposite effect. Finally, biofilm formation was reduced in the *apuA*-deficient mutant and in the presence of glycogen. The authors concluded that ApuA can be regarded as an important virulence factor of *S. suis* promoting hemolysin activity, adherence, and invasion as well as biofilm formation [23]. 

### 3.3. Arginine Deiminase System (ADS)

The *arcABC* operon in *S. suis* encodes the ADS and allows the pathogen to grow under acidic conditions by neutralizing acidification via production of ammonium [116]. The ADS consists of the arginine deiminase (ArcA), the ornithine carbamoyltransferase (ArcB) and the carbamate kinase (ArcC) [89]. Catalyzing the conversion of arginine to ornithine, ammonia as well as CO_2_, the ADS generates energy by the production of ATP [117]. Thereby, it protects bacteria from oxygen and nutrient shortage [118]. *S. suis* is auxotrophic for arginine in CDM [20,119]. Therefore, its survival is dependent on arginine import. The arginine–ornithine antiporter (ArcD) plays an important role in this process as it provides arginine for the ADS which, in turn, is important for *S. suis* fitness and pathogenicity [119]. In *S. suis* the ADS is regulated by the system specific transcriptional regulator ArgR [116]. This contrasts with other bacteria where *argR* regulates genes related to both arginine anabolism and catabolism [120,121,122]. Moreover, the FNR-like protein FlpS of *S. suis* takes part in ADS activation. In an *flps*-deficient mutant the expression of *arcABC* was significantly decreased. *Flps* was shown to be involved in regulating the central carbon and nucleotide metabolism. Oxygen dependent *flps*-mediated activation of arcABC underlines the role of *FlpS* for important adaptation mechanisms to specific in vivo host niches linked to redox conditions [123]. In accordance with these results, the ADS is activated by environmental conditions such as the presence of arginine or glucose and anaerobic conditions [89]. Furthermore, the two-component system (TCS) Ihk/Irr also seems to be involved in ADS regulation. In an *iKR*-deficient mutant strain, ADS was downregulated leading to reduced adherence capacity and stress resistance under acidic conditions [124]. Similarly, in *S. pyogenes* it was also shown that the ADS participates in host cell adhesion [124]. The arginine–ornithine antiporter ArcD provides the ADS of *S. suis* with arginine [119]. This is especially important for intracellular survival of the bacteria, as the generation of ammonium via the ADS can prevent pH drops. Thereby, *S. suis* can resist endosomal acidification inside the host cell. An *arcD-*deficient, an *arcR*-deficient and an *arcABC-*mutant all showed reduced survival inside Hep-2 cells [116,119]. This effect was reduced in Hep-2 cells treated with bafilomycin which prevents endosomal acidification. For that reason, the decreased survival of the mutants is due to a reduced acidic stress resistance [116,119]. 

**Figure 3:**
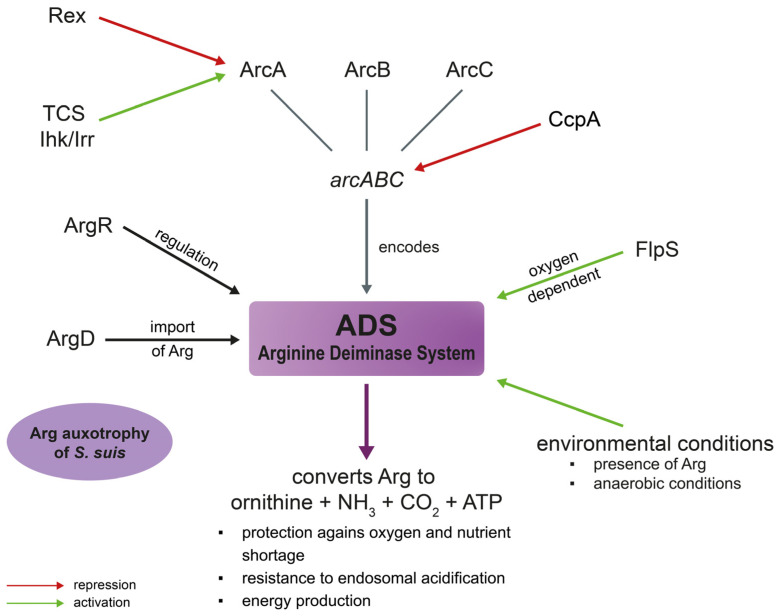
Role of the arginine deiminase system (ADS) in *S. suis* virulence and metabolism. The ADS is regulated by *argR* and encoded by the *arcABC* operon. ArcA can be downregulated by the transcription repressor *rex* [125] and induced by the two-component system Ihk/Irr [124]. The ADS converts arginine to ornithine, ammonia, carbon dioxide and ATP [116,117]. Thereby the ADS contributes to the protection against oxygen and nutrient shortage, energy production as well as resistance to endosomal acidification [116,119]. As *S. suis* is auxotroph for arginine, the transporter ArgD plays an important role in the supply of this amino acid [20,119]. Furthermore, the ADS can be activated by environmental stimuli such as the presence of arginine or anaerobic conditions [89]. Additionally, FlpS can activate the ADS in an oxygen dependent manner [123].

Rex is a redox-sensing regulator involved in metabolism as well as virulence in different bacterial species. A Rex orthologue in *S. suis* was shown to be important for its pathogenicity and stress competence as its absence resulted in reduced virulence and stress resistance [125]. Metabolic pathways associated with central metabolism were altered in the *rex*-deficient mutant. *ArcA* expression was significantly upregulated in the mutant strain. The authors could show that rSsrex was able to directly interact with the *arcA* promoter suggesting a possible function as a transcription repressor of this gene [125]. Figure 3 summarizes the role of the ADS in *S. suis* virulence and metabolism.

### 3.4. Amino Acid Metabolism

Amino acid availability is important for bacteria to synthesize various cell components or for utilization in metabolic pathways [126]. As *S. suis* is auxotroph for different amino acids including tryptophan [20], uptake of these amino acids is of utmost importance. Therefore, e.g., tryptophan transporters are crucial for pathogenesis. Without the substrate-binding protein TrpX of its tryptophan ABC transporter TrpXYZ, *S. suis* is not able to survive in tryptophan-limited conditions [127] or in porcine blood [50]. Therefore, TrpX significantly contributes to nutrient acquisition and bacterial growth during infection. Underlining the important role of ABC transporters in nutrient acquisition they are regarded as important fitness factors that also contribute to bacterial virulence [12].

Another important enzyme for the central carbon catabolism is the PEP carboxylase (*ppc*) which is needed for the biosynthesis of oxaloacetate. Oxaloacetate serves as a precursor for the amino acids aspartic acid and threonine [20]. A *ppc*-deficient mutant of *S. suis* serotype 2 strain 10 showed impaired growth in porcine blood as well as CSF. Although the mutant survived for the tested time, adequate nutrient uptake for normal growth of the pathogen seemed to be missing. Therefore, *ppc* is essential for *S. suis* fitness in the host [20].

## 4. *S. suis* Metabolism and Co-Infections

Respiratory disease is a common problem in the pig industry worldwide. Often pneumonia is not caused by a single pathogen but by an interplay of different viruses and/or bacteria [44].

To the best of our knowledge, a direct study of the metabolic adaptation of *S. suis* to different in vivo niches, co-infected with other pathogens has not been conducted, as *S*. *suis* is also a pathobiont in the respiratory tract of healthy pigs [4]. However, the host immune response as an indirect measure of *S. suis* strategies to establish itself in the host during co-infection with other respiratory pathogens, for example swine influenza virus (SIV) [128,129], porcine reproductive and respiratory syndrome virus (PRRSV) [130,131], and porcine circovirus (PCV) [132] have been investigated and it was found that pre-infection of host cells with the aforementioned pathogens paves the way for *S. suis* infection. Co-infections resulted in higher induction of genes involved in inflammatory response [128,129,130,131,132]. Moreover, co-infection of host cells with SIV was found to facilitate *S. suis* adherence to the virus haemagglutinin protein and further invasion of *S. suis* to the bloodstream due to the structural component of *S. suis* capsule, sialic acid, which allows it as a bacterial virus receptor. The capsular sialic acid of *S. suis* protects capsulated *S. suis* from phagocytosis, and enables the pathogen to invade the respiratory epithelium, spread and induce systemic infection [114,128]. Therefore, we can hypothesize that structural components of *S. suis* in co-infections can indirectly facilitate bacterial metabolic adaptation to certain in vivo niches, making it a dynamic pathogen, capable of a thriving successful infection, with or without co-infecting pathogens.

In addition, co-infections of *S. suis* with SIV, PRRSV or *Bordetella bronchiseptica* were shown to not only promote adherence and invasion but also the cytotoxicity of *S suis* to NPTr cells, porcine alveolar macrophages (PAMs), PCLS or in vivo [114,131,133,134]. On the contrary, an in vitro co-infection experiment with *Glaesserella parasuis* and *S. suis* showed similar adhesion levels of the pathogens in single and co-infection trials [135].

A study by Wang et al. investigated the effect of a co-infection of PCV and *S. suis* in swine tracheal epithelial cells on reactive oxygen species (ROS). The authors showed that the coinfection decreased the activity of NADPH oxidase compared to *S. suis* infection alone [136]. NADPH oxidase is an important ROS generator [137,138]. Therefore, reduced NADPH activity also led to lower ROS concentrations and thereby to an increased intracellular survival of *S. suis* [136]. Changes in enzyme expression and ROS metabolism of the host may also have potential effects on the metabolism of the infecting pathogens.

In conclusion, most of the co-infection studies showed a positive effect on *S. suis* virulence and survival. However, many of these studies have been performed in vitro. Therefore, effects of the host immune system, the residual microflora or environmental factors could not be taken into account. The effects of co-infections on the regulation of metabolic genes need to be addressed in future studies.

## 5. *S. suis* Metabolism and Antibiotic Resistance

Since antibiotic-resistant *S. suis* isolates have increased over the past years, there is an urgent need for new therapeutics. *S. suis* plays an important role as a reservoir for resistance genes [139,140]. Especially macrolides that are often used to treat *S. suis* infections. Therefore, many isolates are resistant against this class of antibiotic [141]. Advances in science and modern technologies like omics approaches demonstrated a link between drug resistance and changes in bacterial metabolism [142,143]. A recent study by Wu et al. investigated the effect of L-serine supplementation on a macrolide resistant *S. suis* isolate [144]. L-serine addition led to both an increased susceptibility to macrolides and a decreased biofilm formation capacity in the resistant strain. Moreover, the authors showed that L-serine supplementation in combination with tylosin administration resulted in an increased level of ROS inside the bacteria leading to enhanced DNA damage [144]. The authors suggest that macrolide resistance in *S. suis* is conferred by an alteration of the serine metabolic pathway along with an inhibition of ROS production [144].

A link between metabolism and antibiotic resistance has also been shown in other bacterial species. Therapeutic efficacy of β-lactam antibiotics was increased in an MRSA *S. aureus* strain by supplementation with d-serine [145]. Exogenous concentrations of alanine or glucose enabled killing of resistant *Edwardsiella tarda* by kanamycin [146]. 

In *S. suis* it was shown that the ABC transporter SatAb was involved in fluoroquinolone resistance by extruding the antibiotics norfloxacin and ciprofloxacin. Although the exact function of this ABC transporter is still unclear, the genetic environment suggests a role in basic metabolism [147]. 

In other bacteria, e.g., *Streptoccocus gordonii* or *S. aureus* CcpA has been shown to be involved in antibiotic resistance. The knock-out of *ccpA* resulted in reduced tolerance towards β-lactam or glycopeptide antibiotics [148,149]. However, whether CcpA also plays a role in antibiotic resistance in *S. suis* needs to be investigated in future studies. In conclusion, all these studies demonstrate that antibiotic resistance mechanisms are closely linked to bacterial metabolism. However, future studies are needed to investigate the role of host metabolites as well as microenvironment specific metabolite generation of the bacteria itself [150]. It needs to be considered that bacterial metabolites can affect each other in the same biological niche [151].

## 6. Summary and Conclusions

In conclusion, metabolic adaptation of *S. suis* to its in vivo niches is a prerequisite for successful survival and establishment of infection. Each niche constitutes a microenvironment with differences in nutrient availability, host defense mechanisms and competing microbiota. *S. suis* adaptation to these varying conditions is reflected in the transcriptomic data obtained in different studies [18,20,49,50]. The analysis of gene transcriptional levels represents a powerful tool to investigate the adaptations and metabolic changes of *S. suis* during infection. In addition, it can reveal valuable insights about the host immune response.

Metabolism regulation also plays an important role in virulence. Mutants deficient in metabolic regulators often showed an attenuated phenotype in infection experiments or survival assays [13,84]. In addition, expression of many virulence factors including *sly* and the capsule of *S. suis* is closely linked to the expression of catabolite control protein A (CcpA) which is involved in sugar metabolism [13,22]. The capsule represents an important protection of *S. suis* against the host immune system [91,92], whereas suilysin(SLY) exhibits cytotoxic effects on various cell types and facilitates invasion [108]. Therefore, they are needed at different stages of infection. Capsule expression is mainly upregulated in the bloodstream to inhibit phagocytosis [8], whereas *sly* expression is induced in different organs, such as the brain and the heart, enhancing colonization and invasion. These host niches represent environments with low glucose concentrations highlighting the link between nutrient availability and virulence [22].

Co-infections of *S. suis* with other pathogens are very common in the pig population [44,152]. They have been shown to promote *S. suis* adhesion, invasion, and virulence to different cell types [114,131,133,134,136]. However, knowledge about the effects on metabolic adaptations of the different pathogens is scarce and needs to be further investigated in future studies. In addition, the role of the existing microbiota during infection needs to be taken into consideration.

Antibiotic resistance (AMR) is a major challenge for human and animal health. The Organisation for Economic Co-operation and Development (OECD) estimates the costs of fighting AMR at up to USD 2.9 trillion by 2050 compared to an AMR-free world [153]. Accordingly, the number of *S. suis* isolates carrying AMR genes has increased over the past decades [139,140]. Importantly, *S. suis* may also spread these genes to other streptococcal species including human pathogens, thereby serving as an AMR reservoir [139]. As metabolism regulation is key for bacteria to survive in the host, it constitutes an interesting target for new therapeutic strategies. Furthermore, metabolism represents an important factor for bacterial persistence [154]. Still today, most of the modes of action of antibiotics concentrate on the synthesis of proteins, folate and the cell envelope or DNA replication [155]. However, different metabolic pathways such as fatty acid metabolism or iron metabolism were also shown to be promising targets for the design of new antibiotics [156,157]. Effects of antibiotics on metabolic pathways can be divided into three main parts: antibiotics can change the metabolism of bacteria resulting in death or growth inhibition; the metabolic state of the pathogen can affect its susceptibility; and the efficacy of antibiotic treatment can be promoted by influencing the bacterial metabolic state [158]. When investigating new targets for antibiotic treatment it is of utmost importance to use targets that do not have a human counterpart or use different catalytic pathways. In addition, bacterial energy metabolism represents a promising target as it differs from usually used AMR sites [159]. Furthermore, ABC transporters which are closely involved in different metabolic processes [160], but also regulatory elements such as T-box riboswitches are regarded as auspicious targets for novel antimicrobials [161,162].

Taken together, metabolic activity of *S. suis* is crucial for its role as a pathobiont in the porcine respiratory tract and, thus also contributes to virulence, Furthermore, mechanisms of metabolic adaptation of *S. suis* in its host should be considered in approaches for new therapeutic strategies.

## Data Availability

Not applicable.
